# Synergistic Roles of Biphasic Ethylene and Hydrogen Peroxide in Wound-Induced Vessel Occlusions and Essential Oil Accumulation in *Dalbergia odorifera*

**DOI:** 10.3389/fpls.2019.00250

**Published:** 2019-03-08

**Authors:** Zhiyi Cui, Zengjiang Yang, Daping Xu

**Affiliations:** Research Institute of Tropical Forestry, Chinese Academy of Forestry, Guangzhou, China

**Keywords:** mechanical wound, essential oil, ethylene, hydrogen peroxide, heartwood, *Dalbergia odorifera*

## Abstract

The heartwood of *Dalbergia odorifera* (*D. odorifera*), named “Jiang Xiang” in traditional Chinese medicine, is highly valuable. Mechanical wounding induced the production of “Jiang Xiang” in *D. odorifera.* Ethylene and hydrogen peroxide (H_2_O_2_) are proposed to play vital roles in wound signaling. However, little is known about the role of ethylene or H_2_O_2_ in the wound-induced formation of vessel occlusions and biosynthesis of “Jiang Xiang” in *D. odorifera*. In this study, the pruning of *D. odorifera* saplings resulted in the synergistic biosynthesis of biphasic ethylene and H_2_O_2_, which was followed by formation of vessel occlusions and “Jiang Xiang” in the pruned stems. In this process, the H_2_O_2_ production stimulated higher biosynthesis of ethylene. Treatments with aminoethoxyvinylglycine (AVG), an inhibitor for ethylene biosynthesis and ascorbate acid (AsA), a scavenger of H_2_O_2_, markedly reduced the production of ethylene and H_2_O_2_, respectively, and the corresponding the percentage of vessels with occlusions (PVO), oil content, and the amount of “Jiang Xiang” formed. These results indicate that ethylene and H_2_O_2_ might be important wound signals in *D. odorifera* that induce vessel occlusions and formation of “Jiang Xiang,” and thus ethylene and H_2_O_2_ might play vital roles in “Jiang Xiang” formation in pruned stems of *D. odorifera*.

## Introduction

**FIGURE 1:**
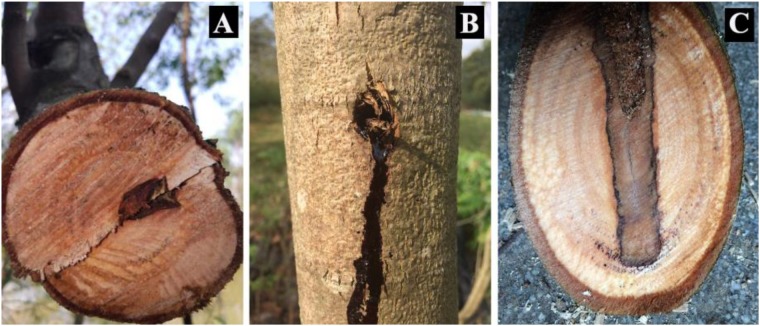
Image description of “JiangXiang” of *Dalbergia odorifera*. **(A)** Represents the natural heartwood (wild “JiangXiang”), which forms very slowly. **(B)** Represents tree trunk suffering from mechanical wound. **(C)** Represents mechanical wound induced “Jiang Xiang” (discolored wood).

*Dalbergia odorifera* T. Chen (*D. odorifera*) is a medium-sized tree from the Leguminosae family, and famous for its heartwood, one of the best rosewoods (Figure 1A) in the world. The heartwood of *D. odorifera*, named “Jiang Xiang” in traditional Chinese medicine, has been widely used as Chinese Pharmacopeia for centuries to stop bleeding, regulate the “Qi,” dissipate blood stasis, and relieve pain (Yukihiro et al., 1985; Cheng et al., 1998; Wang et al., 2000; Sugiyama et al., 2002; Choi et al., 2009; Liu et al., 2017). However, the heartwood of *D. odorifera* forms relatively slowly when trees are more than 6–years-old, and the percentage of heartwood over total stemwood is very small (Ma et al., 2017); thus, study on the promotion of heartwood formation is encouraged (Cui et al., 2017).

Meng et al. (2010) found that mechanical wounding induced the production of “Jiang Xiang” in *D. odorifera* (Figure 1B,C). Similar results were also observed in *Santalum album* (Wei et al., 2000) and *Aquilaria sinensis* (Chen et al., 2012; Wang et al., 2016). Xylem cells destined to form tracheids or vessel members, which provide the conduit for water and mineral transportation, undergo apoptosis (Lucas and Liu, 2017). Vessel occlusions develop in the conduit lumen in response to mechanical wounding in many species for wound sealing and reducing the risk of pathogen intrusion (Saitoh et al., 1993; Dute et al., 1999). Although xylem vessels are primarily dead cells at maturity, they are in contact and communication, especially via pits, with living perivascular parenchyma cells that surround vessels. Perivascular parenchyma cells are active in regulating xylem vessels contents. After wounding or fungal pathogen infection, heartwood substances accumulate in the perivascular parenchyma cells, and are released into the infected vessel lumen to format vessel occlusions for restricting the vessel ingress of the fungus (Zhang et al., 2010).

Ethylene is an important regulator of plant development and growth, and is known to be associated with plant defense (Hou et al., 2013; Savatin et al., 2014). Ethylene production in response to wounding has been demonstrated in a wide range of species. Mechanical wounding induced mandarin (*Citrus unshiu*) (Hyodo and Nishino, 1981), tomato (*Solanum lycopersicum*) (O’Donnell et al., 1996), winter squash (*Cucurbita maxima*) (Kato et al., 2000) and lettuce (*Lactuca sativa)* (Ke and Saltveit, 2010) to regulate the expression of ACS and ACO genes in ethylene synthesis. Sun et al. (2007) found that pruning induced ethylene release and tylose development in grape (*Vitis vinifera*) stems. Van Doorn et al. (1991) also reported that tylose development was reduced in the cut stem of lilac (*Syringa vulgaris*) flowers treated with ethylene inhibitors.

Reactive oxygen species (ROS), which include hydrogen peroxide (H_2_O_2_), superoxide and hydroxyl radicals, have been proposed to play a vital role in wound signaling (Orozco-Cardenas and Ryan, 1999; Ross et al., 2006; Angelini et al., 2008; Mittler et al., 2011). Studies on ROS are always represented by H_2_O_2_ due to its longer half-life (Levine et al., 1994; D’Autréaux and Toledano, 2007). H_2_O_2_ generated in response to wounding can not only be used directly as antimicrobial agents, but also acts as both local and diffusible signal molecules for inducing formation of defense structures, such as callose (Luna et al., 2011) and vessel occlusions (Lorrain et al., 2004; Zhang et al., 2014). H_2_O_2_ can also mediate the elicitor-induced accumulation of secondary metabolites, such as isoflavonoid glyceollin in soybean (*Glycine max*) (Degousee et al., 1994; Guo et al., 1998), p-coumaroyloctopamine in potato tubers (*Solanum tuberosum*) (Matsuda et al., 2001), capsidiol in tobacco (*Nicotiana tabacum*) (Perrone et al., 2003), and sesquiterpenes in *Aquilaria sinensis* (Zhang et al., 2014).

Our field experiment showed that direct injection of ethylene and H_2_O_2_ into the stems of *D. odorifera* resulted in vessel occlusions and formation of “Jiang Xiang,” and it was observed that mechanical wounding induced the production of “Jiang Xiang” in *D. odorifera*. Ethylene and H_2_O_2_ have been proposed to play vital roles in wound signaling, but little information is available on the roles of ethylene and H_2_O_2_ in the wound-induced formation of vessel occlusions and biosynthesis of “Jiang Xiang” in *D. odorifera*. Thus, this study was conducted to investigate whether ethylene or H_2_O_2_ are involved in wounding-induced vessel occlusions and “Jiang Xiang” formation in *D. odorifera*.

## Materials and Methods

### Plant Materials and Experimental Design

Three-year-old saplings of *D. odorifera* were selected to grow in a greenhouse at the Research Institute of Tropical Forestry, Chinese Academy of Forestry, Guangzhou City, Guangdong, China. The saplings were grown under day/night temperatures of 31 ± 3/24 ± 4°C, respectively. The saplings were 1.68 ± 0.32 m in height, with a stem diameter of 2.64 ± 0.21 cm at a height of 10 cm above the ground.

The sapling stems were cut through at 10 cm above the ground in air, water, 0.5 mM aminoethoxyvinylglycine (AVG) (an inhibitor of ethylene biosynthesis) or 1 mM ascorbic acid (AsA) (the special scavenger of H_2_O_2_). The cut ends were soaked in the water, AVG or AsA for 2 h before being exposed to air.

### Ethylene Measurement

Ethylene production in pruned stems was estimated by measuring the ethylene evolved from the cut stem end. To collect gas evolved from the cut, a 5-cm-long rubber tube was attached immediately after each treatment and gas was collected in a 5-mL syringe. Each ethylene sample was collected for half an hour before measurement. The ethylene concentration in the accumulated gas in the syringe was measured at 0.5, 1, 2, 4, 6, 9, 12, 15, 18, 24, 30, 36 and 48 h after treatment, respectively, using an analytical gas chromatograph (Shimazawa, Japan). Five replicate stems were used for the ethylene measurement with each treatment and ethylene production is reported as the concentration in the 5-mL headspace. Because of slight differences in the diameter among the shoots, ethylene concentration data were normalized to a shoot with a 2.64-cm diameter (approximate mean diameter).

The gas chromatograph was equipped with Column SE-54 (30 m × 0.32 mm × 0.25 μm) and column temperature was 70°C. The temperatures of the H^+^-FID flame detector and vaporization chamber were 100°C. Helium was used as carrier gas at a flow rate of 50 mL min^-1^. Hydrogen was used as fuel gas at a flow rate of 60 mL min^-1^. The flow rate of air was 400 mL min^-1^ and the split ratio was 10:1.

### Hydrogen Peroxide Assay

A 1.1-cm-thick stem was collected from the end of each treated stem. Then, a 1-mm-thick section at the end of the cut stem was abandoned, and five 2-mm-thick sections were collected at the positions of 2, 4, 6, 8, and 10 mm from the pruning end. Stem sections were taken at 0, 1, 2, 6, 12, 24 and 36 h after pruning. They were transferred immediately on cutting to liquid nitrogen and stored at -80°C for measurement of endogenous H_2_O_2_. For each sampling, five replicate stems were used for the H_2_O_2_ measurement with each treatment (a total of 140 stems). The samples were immersed in liquid nitrogen and ground to a powder with a pestle and mortar. H_2_O_2_ was analyzed by enzyme-linked immunosorbent assay (ELISA) (Sangon Biotech, Shanghai, China) as described previously with minor modification (Hong et al., 2017). Briefly, the samples, standards, and HRP-labeled detection antibody were added successively in the microporous plate pre-coated with H_2_O_2_ capture antibody, then incubated and thoroughly washed. The substrate tetramethylbenzidine (TMB) was converted to blue by the peroxidase catalysis and finally converted to yellow under the action of an acid. H_2_O_2_ concentrations in the samples were positively correlated with the color intensity. The absorbance (OD value) was measured with a microplate reader at a wave-length of 450 nm to calculate the H_2_O_2_ concentration in the sample.

### Vessel Occlusion Assessment

Sections were prepared from stems as described in section “Hydrogen Peroxide Assay”. For each sampling, five replicate stems were used for assessment of vessel occlusions with each treatment (a total of 140 stems). The samples were collected and fixed in formalin acetic acid-alcohol (FAA) for assessment of vessel occlusions at 0, 1, 2, 3, 4, 5 and 6 weeks, respectively. The percentage of vessel occlusions (PVO) was assessed as previously described (Sun et al., 2007). Briefly, transverse sections (20 μm in thickness) were prepared with a sliding microtome (Leica RM2255, Germany). The sections were temporarily mounted with a cover slip in water, then observed under light microscopy (Olympus BX51, Japan) equipped with a digital camera (Pixera Pro 600ES, United States). Five areas, each containing 20–30 vessels, were chosen randomly for analysis. The analysis of each replication (cut stem) included 100–150 vessels.

### Essential Oil Assessment

The samples were collected as mentioned above for assessment of essential oil at 0, 1, 2, 3, 4, 5, and 6 weeks, respectively. For each sampling, five replicate stems were used for assessment of essential oil with each treatment (a total of 140 stems). The samples were immediately immersed in liquid nitrogen and ground to a powder with a pestle and mortar. For each treatment, 1 g powdered stem samples were weighed, immersed in 30 ml petroleum ether and shaken for 24 h. After filtration and concentration (Concentrator 5301, Eppendorf, Germany), essential oil was obtained and the oil content was also calculated.

Essential oil from the 6 week harvest was used for GC-MS with an Agilent 6890 N-5975 I system with an Innowax DB-5MS column (30 m × 0.25 mm, 0.25 μm film thickness). Helium was used as carrier gas at a flow rate of 1 mL min^-1^. Oven temperature was programmed to 70°C for 1 min, raised to 250°C at a rate of 8°C min^-1^, and kept constant at 250°C for 15 min. Mass spectra were recorded at 70 eV with the mass range *m/z* 35 to 450. The identification of essential oil components was done by computer matching against NIST and Wiley GC-MS Library or comparing the retention times of oil components with standard samples.

### Statistical Analysis

Data were calculated based on combined averages from five individual saplings (*n* = 5) per treatment. The plots for the graphs were generated in SigmaPlot 10.0 (Systat, United States). The significance of differences among treatments was evaluated with Duncan’s multiple range tests using the data processing software SPSS 17.0 (IBM, NY, United States).

## Results

### Wounding Induced Enhanced Ethylene Production

**FIGURE 2:**
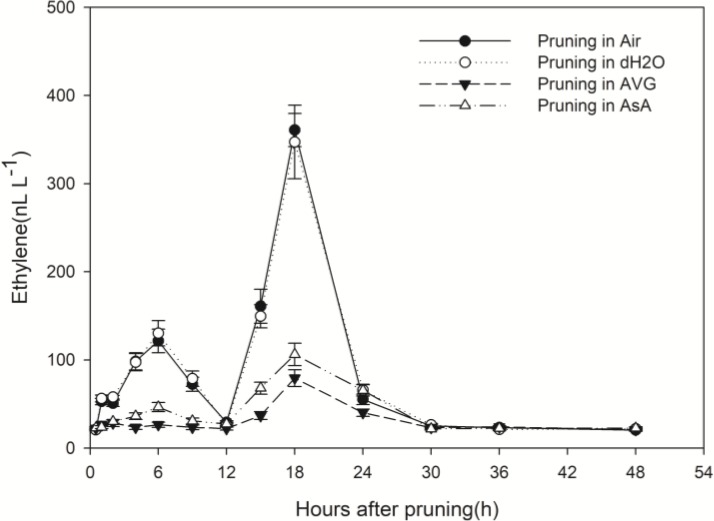
Effect of different pruning treatments on ethylene concentrations. Ethylene concentrations were normalized to a cut surface with a 2.64-cm diameter (approximate mean diameter 10 cm above ground). Error bars represent ± S.D, *n* = 5.

Whether stems were pruned in air or in water, production of ethylene increased in response to wounding. Ethylene production increased in a biphasic manner with peaks at about 6 h and 18 h after pruning and an intervening decline to the initial level at 12 h (Figure 2). The second peak was about 3-times greater than the first peak and 15-times greater than the initial concentration. By 30 h after pruning, the ethylene concentration was again at the initial level where it remained with a slight diurnal oscillation (Figure 2).

In contrast, the pattern of ethylene production from the pruned end of stems treated with AVG or with AsA was dramatically different from the treatments in air and water. These two treatments almost eliminated the first rise in ethylene concentration; the second increase was greatly reduced too. The second peak value was 360.82 and 347.30 nL L^-1^ in stems pruned in air and in water, respectively, while it was only 79.41 and 106.22 nL L^-1^ in stems pruned in AVG and in AsA, respectively (Figure 2). Ethylene production induced by wounding was greatly suppressed in the presence of AVG and AsA.

### Wounding Induced Enhanced H_2_O_2_ Production

**FIGURE 3:**
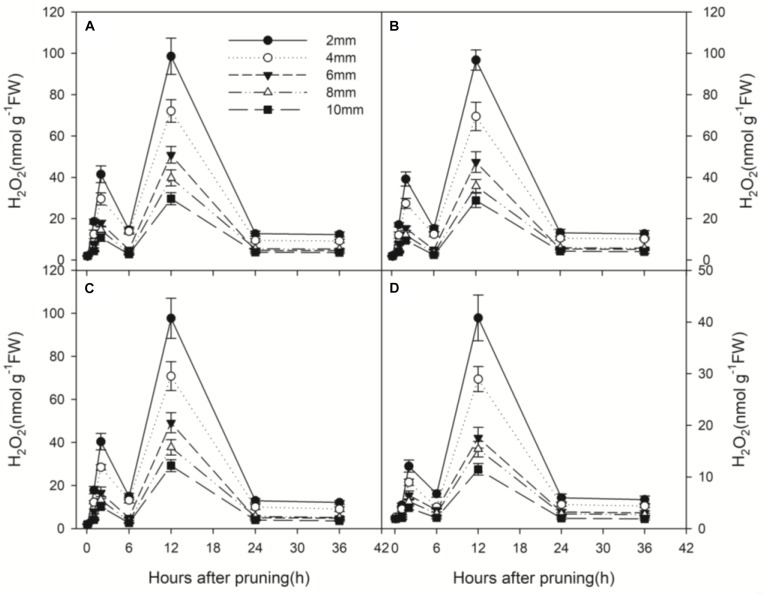
Temporal progress in H_2_O_2_ concentration in xylem tissue of wounded stems at different depths (2, 4, 6, 8, and 10 mm) from the cut surface of stems pruned in air **(A)**, water **(B)**, AVG **(C)** and AsA **(D)**. Error bars represent ± S.D, *n* = 5.

Wound-induced H_2_O_2_ production was measured from 0 to 36 h after pruning at 5 depths from the wound surface. H_2_O_2_ also produced in a biphasic manner, peaked at about 2 h and 12 h after pruning and declined to the initial level at 6 h and 24 h (Figure 3). The second peak of H_2_O_2_ production was about 2.5 times of the first peak and almost 40 times higher than the initial concentration. Generally, H_2_O_2_ production decreased with distance from the wound surface (Figure 3). The peak in H_2_O_2_ concentration at 12 h after pruning decreased with distance from the wounded surface, ranging from 98.60 nmol g^-1^ FW at 2 mm to 29.65 nmol g^-1^ FW at 10 mm (Figure 3A).

Wound-induced H_2_O_2_ production of the treatment with AsA (Figure 3D) was greatly reduced compared to the treatments in air and water (Figure 3A,B). However, no significant difference was found in H_2_O_2_ production between the treatments in air, water and AVG (Figure 3A–C). The peak in H_2_O_2_ concentration at 12 h after pruning was 98.60, 96.75 and 97.68 nmol g^-1^ FW in stems pruned in air, water and AVG, respectively. By contrast, it was only 40.82 nmol g^-1^ FW in stems pruned in AsA (Figure 3). Thus, wound-induced H_2_O_2_ production was greatly suppressed in the presence of AsA, but not under the influence of AVG.

### Wound-Induced Vessel Occlusions and Essential Oil at Different Depths From the Cut Surface

**FIGURE 4:**
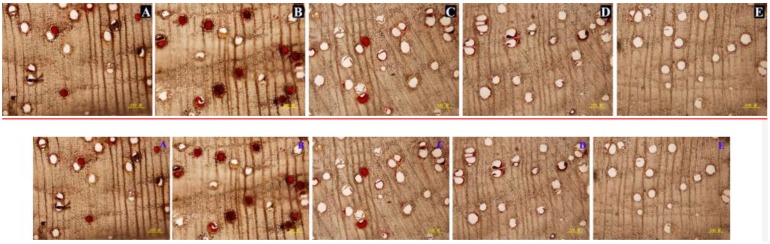
Vessel occlusions of transverse sections at different depths from the cut surface at 6 weeks after pruning. **(A–E)** Represents transverse sections at 2, 4, 6, 8, and 10 mm from the cut surface, respectively. Scale bars = 200 μm.

**FIGURE 5:**
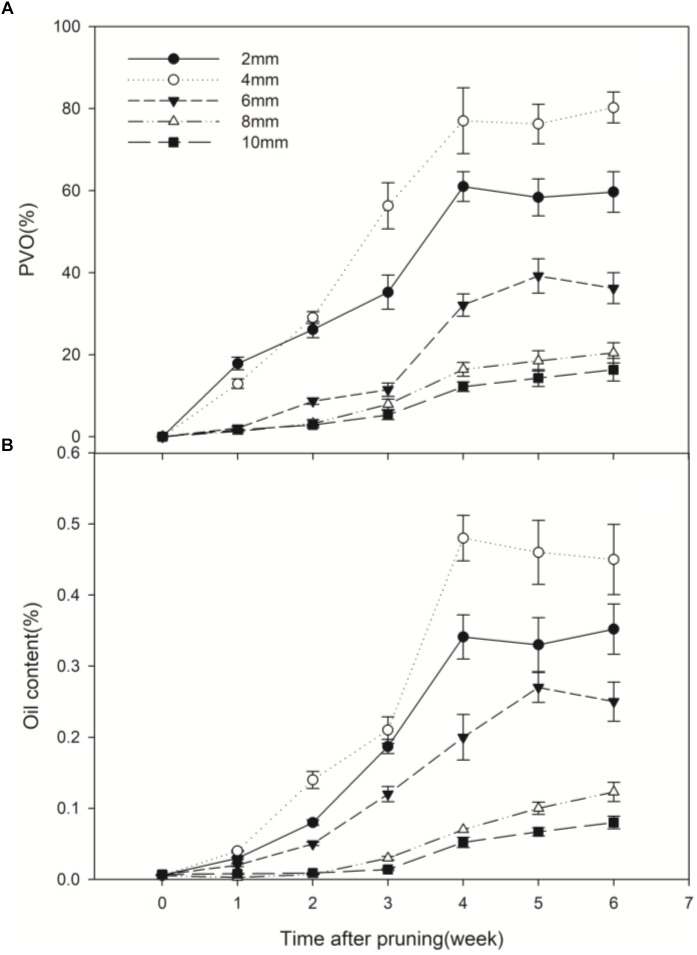
Temporal progress in pruning-induced percentage of vessel occlusions **(A)** and essential oil content **(B)** at different depths from the cut surface in stems pruned in air. PVO, the percentage number of vessels with occlusions. Error bars represent ± S.D, *n* = 5.

Prior to pruning, the *D. odorifera* stems had essentially no occlusions. After pruning in air, occlusions were observed in the vessel lumens, and continued production of vessel occlusions sealed some vessel lumens. A significant difference in vessel occlusions was observed between depths from the cut surface (Figure 4). The percentage of vessels with occlusions (PVO) kept increasing in the first 4 weeks after pruning. After that, the changes were small. PVO increased much faster at 2, 4 and 6 mm compared to 8 and 10 mm, and PVO at 4 mm increased at a faster rate than other distances. Four weeks after pruning, the PVO at 2 and 4 mm sections reached 60.98 and 77.01%, respectively, while they were only 16.43 and 12.20% for 8 and 10 mm sections, respectively (Figure 5A). The pattern of essential oil content was almost synchronous with that of PVO (Figure 5B).

Nine major volatiles were identified in the essential oil extracted from the samples at different depths from the wound surface. However, none of these volatiles was found in the control samples (stems prior to pruning). The relative amount of volatiles was highest at 4 mm, and decreased significantly above and below this depth (Table 1). These results showed that wounding induced the most vessel occlusions and essential oil at 4 mm from the cut surface.

**Table 1:** Chemical composition and relative amounts of essential oil from the samples at different depths from the cut surface in stems pruned in air after 6 weeks treatment.

No.	Compound name	Relative amount (%)					
		2 mm	4 mm	6 mm	8 mm	10 mm	CK
1	(E)-.beta.-Famesene	0.02 ± 0.00b	0.05 ± 0.00a	–c	–c	–c	–c
2	.alpha.-Farnesene	0.25 ± 0.03a	0.28 ± 0.03a	0.07 ± 0.01b	–c	–c	–c
3	2-Cyclohexen-1-ol, 2-methyl-5-(1-methylethenyl)-, *cis*-	0.36 ± 0.04b	1.58 ± 0.12a	0.32 ± 0.03b	–c	–c	–c
4	1,5-Heptadiene, 3,3-dimethyl-, (E)-	1.01 ± 0.08b	1.49 ± 0.16a	0.67 ± 0.05c	–d	–d	–d
5	2-Isopropenyl-5-methylhex-4-enal	0.42 ± 0.02b	0.67 ± 0.07a	0.38 ± 0.04b	0.15 ± 0.01c	–d	–d
6	2-Cyclohexene-1-carboxaldehyde, 2,6,6-trimethyl-	0.7 ± 0.05b	0.92 ± 0.06a	0.67 ± 0.04b	0.41 ± 0.02c	–d	–d
7	Citronellol	0.05 ± 0.01b	0.26 ± 0.03a	–c	–c	–c	–c
8	Butanoic acid, 3-hexenyl ester, (Z)-	1.22 ± 0.09b	1.76 ± 0.21a	1.30 ± 0.09b	0.75 ± 0.05c	–d	–d
9	Nerolidol	0.23 ± 0.01b	0.56 ± 0.03a	0.08 ± 0.01c	–d	–d	–d


*The same letters indicate that the difference is not significant at the 0.05 level (p > 0.05, Duncan’s), “–” means not detected. All data are shown as mean ± standard deviation.*

### Wound-Induced Vessel Occlusions and Essential Oil in Stems of Different Treatments

**FIGURE 6:**
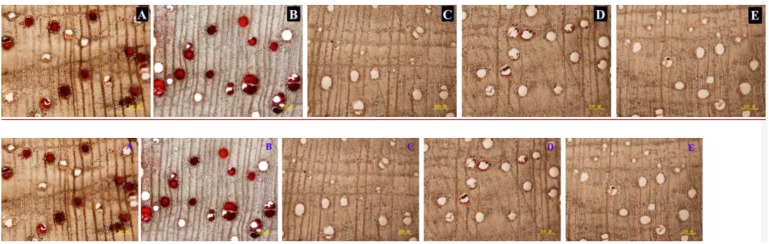
Vessel occlusions of transverse sections at 4 mm from the cut surface in stems of different treatments at 6 weeks after pruning. **(A–E)** Represents transverse sections of treatment pruned in air, dH_2_O, AVG or AsA and prior to pruning, respectively. Scale bars = 200 μm.

**FIGURE 7:**
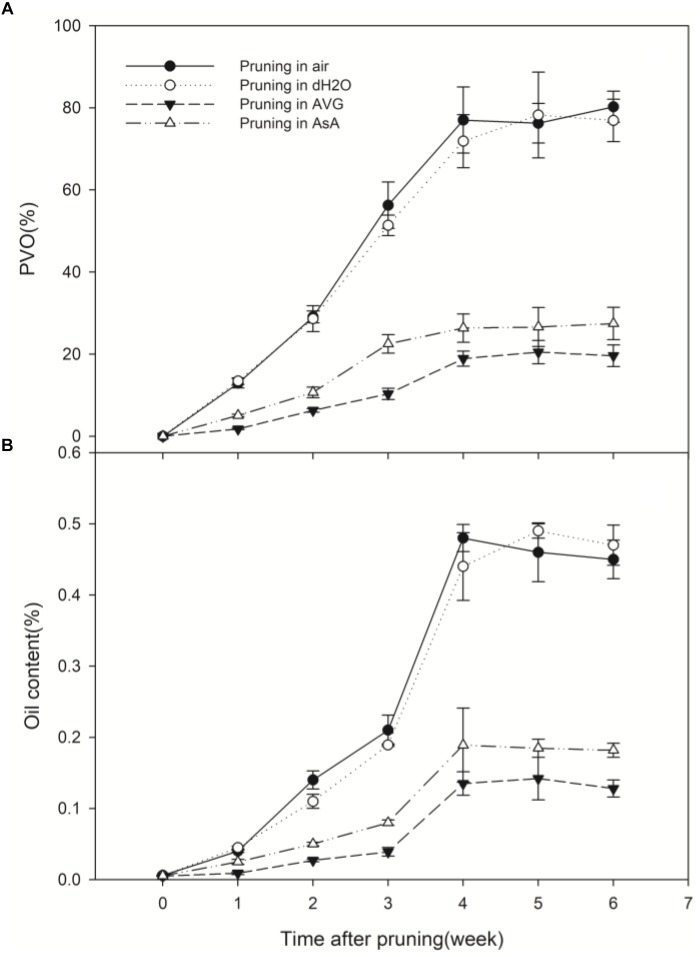
Temporal progress in pruning-induced percentage of vessel occlusions **(A)** and essential oil content **(B)** at 4 mm from the cut surface in stems pruned in air, water, AVG or AsA. PVO, the percentage number of vessels with occlusions. Error bars represent ± S.D, *n* = 5.

In the stems pruned in air or water, occlusions were observed in a large number of vessels in secondary xylem (Figure 6A,B), while occlusions were absent in most of the vessels in the stems treated with AVG or AsA (Figure 6C,D). The PVO and essential oil content were markedly affected by the AVG and AsA treatments. At 6 weeks after pruning, PVO at 4 mm section of stems treated in air, water, AVG or AsA reached 80.24, 76.92, 19.6 and 27.46%, respectively (Figure 7A). Consequently, the essential oil content was 0.45, 0.47, 0.13 and 0.18% for stems treated in air, water, AVG or AsA, respectively (Figure 7B). These results indicated that suppression of wound-induced ethylene or H_2_O_2_ production significantly reduced vessel occlusions and essential oil content.

The kind and quantity of the volatiles in the treatment pruned in AVG or AsA also decreased significantly compared to the treatments pruned in air and water (Table 2). Similar volatiles were detected in the treatments pruned in air and water. Nine volatiles identified in wild “Jiang Xiang” was found in both treatments pruned in air and water and relative amounts of them were not statistically different. However, the volatiles of the treatment pruned in AVG or AsA were significantly different from the treatments in air and water. Only two and four volatiles were identified in stems pruned in AVG and AsA, respectively, which had fewer compounds than those in air and water (Table 2).

**Table 2:** Chemical composition and relative amount of essential oil at 4 mm from the cut surface in stems pruned in air, water, AVG or AsA after 6 weeks treatment and prior to pruning (CK).

No.	Compound name	Relative amount (%)					
		Pruning in air	Pruning in dH_2_O	Pruning in AVG	Pruning in AsA	CK
1	(E)-.beta.-Famesene	0.05 ± 0.01a	0.06 ± 0.01a	–b	–b	–b
2	.alpha.-Farnesene	0.28 ± 0.03a	0.32 ± 0.03a	–b	–b	–b
3	2-Cyclohexen-1-ol, 2-methyl-5-(1-methylethenyl)-, cis-	1.58 ± 0.13a	1.67 ± 0.15a	0.12 ± 0.01b	0.26 ± 0.01b	–c
4	1,5-Heptadiene, 3,3-dimethyl-, (E)-	1.49 ± 0.09a	1.39 ± 0.11a	–c	0.11 ± 0.02b	–c
5	2-Isopropenyl-5-methylhex-4-enal	0.67 ± 0.04a	0.71 ± 0.06a	–b	–b	–b
6	2-Cyclohexene-1-carboxaldehyde, 2,6,6-trimethyl-	0.92 ± 0.10a	0.81 ± 0.09a	–c	0.53 ± 0.03b	–c
7	Citronellol	0.26 ± 0.01a	0.23 ± 0.01a	–b	–b	–b
8	Butanoic acid, 3-hexenyl ester, (Z)-	1.76 ± 0.12a	1.89 ± 0.15a	0.53 ± 0.07b	0.69 ± 0.05b	–c
9	Nerolidol	0.56 ± 0.03a	0.48 ± 0.04a	–b	–b	–b


*The same letters indicate that the difference is not significant at the 0.05 level (p > 0.05, Duncan’s), “–”means not detected. All data are shown as mean ± standard deviation.*

## Discussion

One of the prominent phenomena of plants in the response to external stimuli is the production pattern of ROS. Biphasic H_2_O_2_ production with first a minor burst followed by a second major burst has been reported in several plants. In this study, there were two bursts of H_2_O_2_ production in *D. odorifera* stems after wounding, which was also observed in Arabidopsis and ryegrass (Le Deunff et al., 2004; Song et al., 2006). In addition, there were less vessel occlusions and amount of “Jiang Xiang” at 2 mm depth or at ≥ 6 mm due either high or low concentrations of H_2_O_2_ resulting in parenchyma cells necrosis. The medium H_2_O_2_ concentration at 4 mm depth induced the most vessel occlusions and “Jiang Xiang.” This result was consistent with the fact that H_2_O_2_ plays a dual role in plants: it acts as a signal molecule at low concentrations and it leads to necrosis at high levels (Gill and Tuteja, 2010).

Along the same pattern, ethylene production also burst twice in response to wounding. A similar pattern of wound-induced ethylene production was described in grape (Sun et al., 2007). Also, the hormone jasmonic acid was induced in a biphasic manner in response to wounding in pea seedlings (Yang et al., 2009). When wounding was conducted in air and in water, similar ethylene or H_2_O_2_ production was observed. It is understandable that treatments with ethylene or H_2_O_2_ inhibitors significantly inhibited ethylene or H_2_O_2_ production in wounded stems, respectively. In contrast, it is intriguing thing that AsA, the special scavenger of H_2_O_2_, also greatly inhibited ethylene production. This result indicated that the reduction in H_2_O_2_ inhibited ethylene production induced by wounding. Meanwhile, wound-induced H_2_O_2_ production was not suppressed in the presence of AVG, a specific inhibitor for ethylene biosynthesis. In addition, the first H_2_O_2_ burst at 2 h (Figure 3) and a significant ethylene production peak at 6 h (Figure 2) were observed. Thereafter, the second H_2_O_2_ burst and another ethylene production peak occurred at 12 h and 18 h, respectively (Figure 2, 3). These results suggest that mechanical wounding induced biphasic H_2_O_2_ and ethylene production and each H_2_O_2_ burst was followed by an ethylene production peak. Hence, it appears that H_2_O_2_ production was required for ethylene production. Similar biphasic ROS or ethylene production was observed in plants subjected to other stresses such as ozone (Xie et al., 2011) and pathogen infection (Wi et al., 2012). After inoculation with *Pseudomonas syringae*, ethylene production in tobacco leaves followed a biphasic pattern reminiscent of H_2_O_2_ production (Mur et al., 2008). Wi et al. (2012) further provided evidence that a pathogen-induced oxidative burst was required as an upstream regulator of ethylene production in both phases. They suggested that the rapid transient increases in ROS generation followed by ROS-induced ethylene production act as a determinant of a hypersensitive response, whereas the later massive ROS burst and subsequent massive ethylene production act as a positive determinant of pathogen expansion and cell death in compatible interactions. Wi et al. (2012) also hypothesized that the late massive ROS burst stimulated higher biosynthesis of ethylene, which could promote disease susceptibility. In this process, the biphasic production of ethylene and ROS might be regulated by a self-amplifying loop (Wi et al., 2012). The issue of ROS signal specificity has recently received considerable attention. One possibility is that the specific features of ROS signaling could be perceived and decoded into specific responses, which determine gene expression patterns (Mittler et al., 2011). Despite these results, detailed studies on biphasic H_2_O_2_ or ethylene production have not been performed.

Nine major volatiles in wild “Jiang Xiang” (Ma et al., 2017) were identified in the essential oil extracted from stems pruned in air or water, none of which was found in the control samples (stems prior to pruning). As suppression of wound-induced ethylene or H_2_O_2_ production markedly reduced vessel occlusions and oil content, the kind and quantity of the volatiles in the treatments pruned in AVG or AsA decreased significantly compared to the treatments pruned in air and water. These results showed that wound-induced ethylene and H_2_O_2_ production were accompanied by vessel occlusions and accumulation of “Jiang Xiang” in pruned stems of *D. odorifera*. In other words, ethylene and H_2_O_2_ played vital roles in wound-induced vessel occlusions and accumulation of “Jiang Xiang” in *D. odorifera*.

This study demonstrated that wound-induced ethylene and H_2_O_2_ played vital roles in vessel occlusions and accumulation of “Jiang Xiang” in *D. odorifera*. They might regulate vessel occlusions and formation of “Jiang Xiang” in pruned stems of *D. odorifera*. Mechanical wound or fungal pathogen infection can induce structural barriers (including callose deposition, cell wall thickening and vessel occlusions), phytoalexins production, and induced expression of defense-related genes (Ahuja et al., 2012). For example, after fungal infection, rapid vessel occlusions by tyloses and formation of fungitoxic terpenoid aldehydes were observed in resistant cotton (Zhang et al., 1993; Daayf et al., 1997). It is hypothesized that pruning caused the production of wound signals (ethylene and H_2_O_2_). In this process, the H_2_O_2_ production stimulated higher biosynthesis of ethylene. Then synergistic biosynthesis of biphasic ethylene and H_2_O_2_ resulted in the formation of structural barriers (vessel occlusions) and formation of phytoalexins (“Jiang Xiang”) that may contribute to physical barriers and chemical inhibition of microbes within vessels to prevent their spread. The PVO and amount of secondary substances increased with duration after pruning, and ultimately “Jiang Xiang” was formed in pruned stems of *D. odorifera*.

## Conclusion

It was concluded that mechanical wounding resulted in the synergistic biosynthesis of biphasic H_2_O_2_ and ethylene, vessel occlusions and “Jiang Xiang” formation in *D. odorifera*. In this process, the H_2_O_2_ production stimulated higher biosynthesis of ethylene. Suppression of ethylene or H_2_O_2_ reduced the amount of vessel occlusions and “Jiang Xiang” production significantly. These results indicate that ethylene and H_2_O_2_ play vital roles in vessel occlusions and formation of “Jiang Xiang” in *D. odorifera* in response to mechanical wounding, and thus ethylene and H_2_O_2_ could be applied to induce formation of “Jiang Xiang” in *D. odorifera*. Further study is needed to explore the molecular mechanism of ethylene and H_2_O_2_ regulation on formation of “Jiang Xiang” in *D. odorifera*.

## Data Availability

All datasets generated for this study are included in the manuscript and/or the supplementary files.

## Author Contributions

ZC wrote the manuscript. ZY helped in the experiments. DX revised the manuscript.

## Conflict of Interest Statement

The authors declare that the research was conducted in the absence of any commercial or financial relationships that could be construed as a potential conflict of interest.
